# Sutureless versus conventional deep sclerectomy for management of open angle glaucoma

**DOI:** 10.1007/s00417-022-05910-4

**Published:** 2022-11-26

**Authors:** Mohamed Sabry Kotb, Shahenda Ahmed El Gharbawy, Ahmed Mostafa Abdelrahman, Hebatalla Samir Makled

**Affiliations:** Ophthalmology Department, Faculty of Medicine, Kasr Al Ainy Hospital, Cairo University, Cairo, 11562 Egypt

**Keywords:** Sutureless deep sclerectomy, Conventional deep sclerectomy, Open-angle glaucoma, Intraocular pressure, Bleb

## Abstract

**Introduction:**

To compare sutureless deep sclerectomy to conventional deep sclerectomy regarding their lowering effect on intraocular pressure (IOP) in cases with open-angle glaucoma.

**Methods:**

This is a prospective interventional randomized comparative study that included 60 eyes of 50 patients with open-angle glaucoma (OAG) who were indicated for surgical intervention. Patients were recruited from the glaucoma subspecialty clinic of the Cairo University teaching hospital and were divided into two groups: group A (underwent sutureless deep sclerectomy) and group B (underwent conventional deep sclerectomy).

**Results:**

Both surgeries showed significant reduction of IOP all through the study period: in group A, mean reduction was 71.37%, 53.35%, 50.3%, and 44.33% at 1st day, 1 month, 3 months, and 6 months respectively, and in group B, mean reduction was 57.62%, 40.63%, 37.41%, and 31.68% at 1st day, 1 month, 3 months, and 6 months, respectively. Comparison between percentage of reduction in both groups showed no statistically significant difference. Also, use of anti-glaucoma medications dropped significantly at 6 months postoperatively in both groups with no significant difference between the 2 groups. Regarding reported complications, 12.9% in group A and 10.3% in group B presented with non-serious complications. One month postoperatively, UBM detected non-functioning blebs in 6.4% of group A and 3.4% in group B. Other cases with non-functioning blebs were detected at 3 and 6 months postoperatively, and all cases were managed.

**Conclusion:**

Sutureless deep sclerectomy seems to be a safe and effective modification, with significant IOP reduction in POAG.


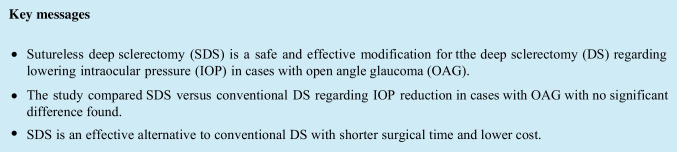


## Introduction

Deep sclerectomy (DS) is a non-penetrating glaucoma surgery that has emerged as a competitor to the standard trabeculectomy which is safer with comparable results regarding intraocular pressure (IOP)-lowering effect in cases with open-angle glaucoma (OAG). DS entails non-penetration of the anterior chamber through leaving an intact trabeculo-Descemetic membrane (TDM) that acts as barrier to prevent marked postoperative hypotony that follows trabeculectomy [1].

The presence of intra-scleral lake is a unique feature of DS, and its dimensions can be correlated with postoperative IOP in cases with OAG [2]. Different modifications and adjuvants have been introduced to the original technique to maintain the presence of intrascleral lake and prevent bleb failure [3–6].

Sutureless deep sclerectomy (SDS) is a relatively recent modification to DS that converts the conventional surgery to be completely sutureless with no sutures at the scleral flap or the conjunctival edges. This technique is supposed to decrease complications like foreign body reaction, excessive tissue trauma, and local irritation due to the use of sutures. It has shorter operation time, there is no need for postoperative suture removal, and it is more economic than the conventional one [7].

The aim of this study is to compare sutureless deep sclerectomy to conventional deep sclerectomy regarding their lowering effect on IOP in cases with open-angle glaucoma.

## Methodology

This is a prospective interventional randomized comparative study that compared sutureless versus conventional deep sclerectomy as a primary surgical management for open-angle glaucoma (OAG). The study included 60 eyes of 50 patients with OAG who were indicated for surgical intervention. The study patients were recruited from the Glaucoma Subspecialty Clinic of the Cairo University Teaching Hospital during the period between September 2018 and April 2020. Patients were divided into two groups: group A (they underwent sutureless deep sclerectomy) and group B (underwent conventional deep sclerectomy). Randomization was done in the outpatient clinic where the recruited cases were given numbers from 1 to 60 with eyes with odd numbers added to group A and those with even numbers added to group B. All patients signed a written consent to participate in the study and to approve sharing of the study data after thorough explanation of the surgical technique and its expected drawbacks. The study was performed according to the tenets of the Declaration of Helsinki and was approved by the Scientific Committee of the Ophthalmology Department of the Faculty of Medicine, Cairo University.

Inclusion criteria for the study were patients with open-angle glaucoma who were indicated for surgical intervention due to failure to control IOP despite the use of maximally tolerated medical treatment, non-adherence, or intolerance to anti-glaucoma medications. Exclusion criteria were angle closure glaucoma, uveitic glaucoma, neovascular glaucoma, and history of previous glaucoma surgery.

The selected cases were diagnosed as OAG on basis of clinical examination, visual field examination, and retinal nerve fiber layer analysis with spectral domain-optical coherence tomography (SD-OCT) according to the European Glaucoma Society guidelines [8].

History was taken from each patient with emphasis on family history, previous ocular surgeries, and number and duration of glaucoma medications used. Then, each patient underwent full ophthalmological examination including corrected distance visual acuity (CDVA) using Snellen chart and recorded in Log MAR, anterior segment examination using Slit lamp, posterior segment examination using slit lamp biomicroscopy and 90-D lens (Volk, VOLK Optical; Mentor, Ohio, USA), IOP measurement using Goldmann applanation tonometer GAT (HAAG-STREIT AT 900, Edinburgh Way, Harlow, UK), and gonioscopy using three-mirror Goldmann contact lens (Volk, VOLK Optical; Mentor, Ohio, USA) with grading of the angle according to the Scheie classification system [9]. Investigations included SD-OCT retinal nerve fiber layer assessment and visual field testing.

**Fig. 1:**
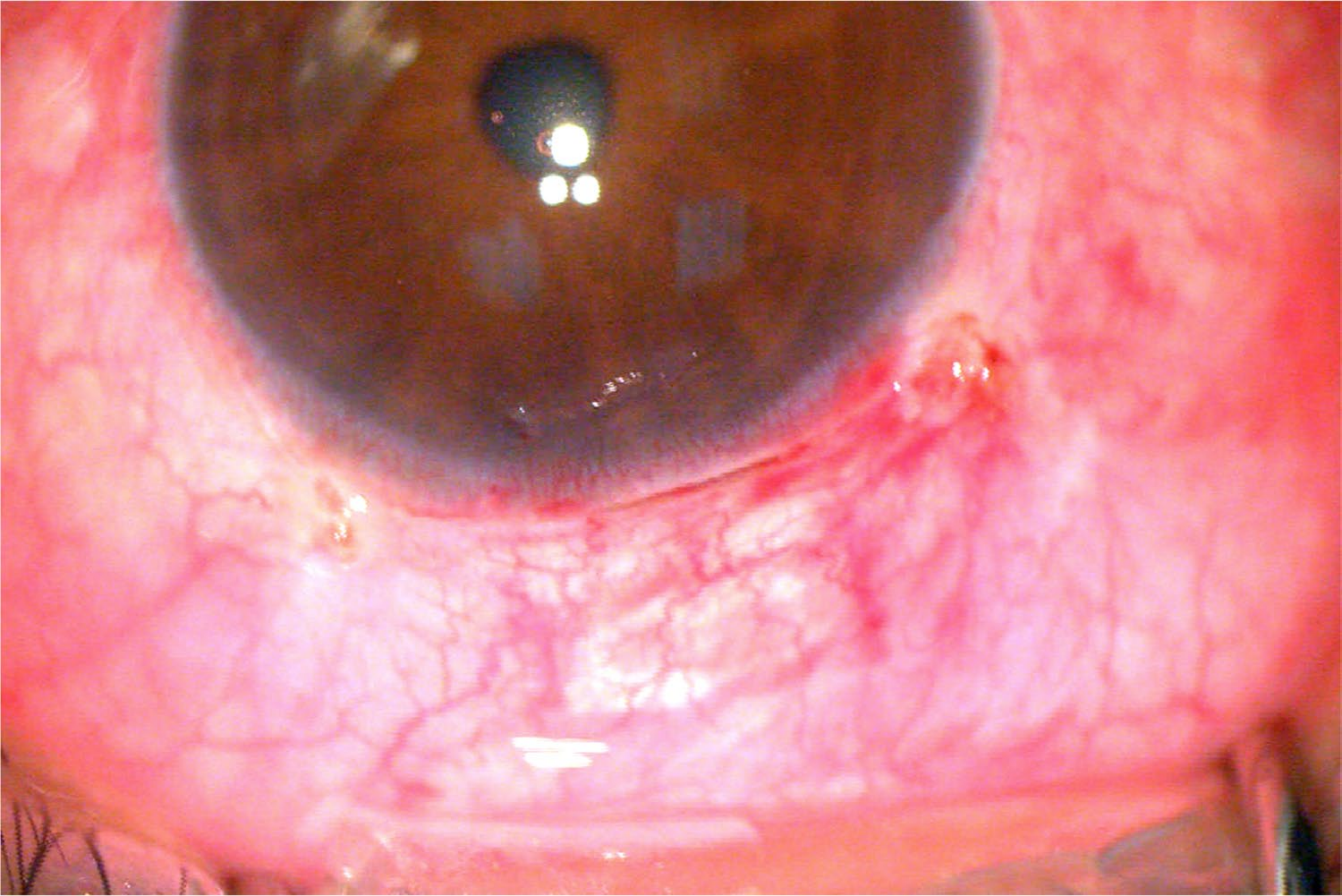
One eye of sutureless deep sclerectomy group at the end of surgery with the scleral flap left un-sutured under the conjunctival flap that was re-approximated with 30–40% diathermy

All patients underwent deep sclerectomy under peri-bulbar anesthesia by the only 2 surgeons (A. M. A. and M. K.); each surgeon has done equal number of patients of the 2 groups. A superior corneal traction suture was taken using 7–0 Vicryl. The conjunctiva was dissected by doing fornix-based flap followed by minimal diathermy application to cauterize the bleeders. Superficial scleral flap (4 × 3 mm) was dissected using crescent blade, and the dissection was carried out anteriorly into the peripheral 2 mm of clear cornea. Mitomycin C (MMC) (Kyowa Co., Hakko Kirin, Japan) with concentration (0.4 mg/ml) was applied for 2 min underneath the superficial scleral flap before dissecting the deep one and that under the dissected conjunctiva, and then washed by 20 ml of balanced salt solution (BSS). Paracentesis was done using microvitreoretinal (MVR) blade. The same crescent blade was used to dissect the deep scleral flap just inside the margins of the superficial one with deroofing of the Schlemm’s canal and exposing the trabeculo-Descemetic membrane. The deep flap was then excised using fine scissors, and the percolation was tested to be adequate with cellulose microsponge. In group A, the superficial scleral flap was left un-sutured to freely float and return in position, and the conjunctival edges were re-approximated with low-power diathermy (30 to 40%) (Fig. 1).

In group B, the superficial scleral flap was sutured in place with two 10/0 Nylon sutures, and the conjunctiva was sutured in place using 8/0 polyglactin sutures. The presence of conjunctival leak was tested by using a dry cellulose sponge. An ointment with combination of steroid and antibiotic was applied to the ocular surface at the end of the surgery.

Follow up was done at the 1st day, 1 week, 1 month, 3 months, and 6 months postoperatively. In each visit, the eye was examined for IOP, depth of anterior chamber with any signs of inflammation, any conjunctival leak, and changes in the fundus especially choroidals. Ultrasound biomicroscopy (UBM) was done for all patients at 1 month and 6 months postoperatively.

Complete success was defined if IOP ranged from 6 to 18 mmHg throughout the follow-up period without use of glaucoma medications, while qualified success was defined if IOP ranged from 6 to 18 mmHg with anti-glaucoma medications. Use of anti-glaucoma medications or interventions done for reducing IOP was reported at different spots of follow up.

### Statistical analysis

Data were coded and entered using the statistical package for the Social Sciences SPSS (IBM, Armonk, NY) version 24. Data was summarized using mean, standard deviation, median, minimum, and maximum in quantitative data and using frequency (count) and relative frequency (percentage) for categorical data. Comparisons between quantitative variables were done using the non-parametric Mann–Whitney test. For comparison of serial measurements within each patient, the non-parametric Wilcoxon signed rank test was used. For comparing categorical data, Chi square (*χ*^2^) test was performed. Exact test was used instead when the expected frequency is less than 5. *P* values less than 0.05 were considered as statistically significant.

## Results

Sixty eyes of 50 patients with medically uncontrolled OAG were divided into two groups: group A (sutureless deep sclerectomy included 31 eyes of 27 patients) and group B (conventional deep sclerectomy included 29 eyes of 23 patients). The mean age of the study subjects was 46.45 ± 11.50 years. Twenty-three patients were males (46%), and 27 were females (54%). Demographic and preoperative data of the two groups are displayed in Table 1.

**Table 1:** Demographic and preoperative data

	Group A		Group B		*P* value
Mean age	43.48 years ± 13.57		49.32 years ± 8.38		0.098
Sex	Males	Females	Males	Females	
	14 patients (51.9%)	13 patients (48.1%)	9 patients (39.14%)	14 patients (60.86%)	
Preoperative mean IOP	31.68 mmHg ± 8.85		25.11 mmHg ± 7.35		0.003
Preoperative mean no. of medications	2.91 ± 1.1		2.21 ± 0.418		0.001
Preoperative mean CDVA by LogMar	0.68 ± 0.17		0.65 ± 0.11		0.78

**Fig. 2:**
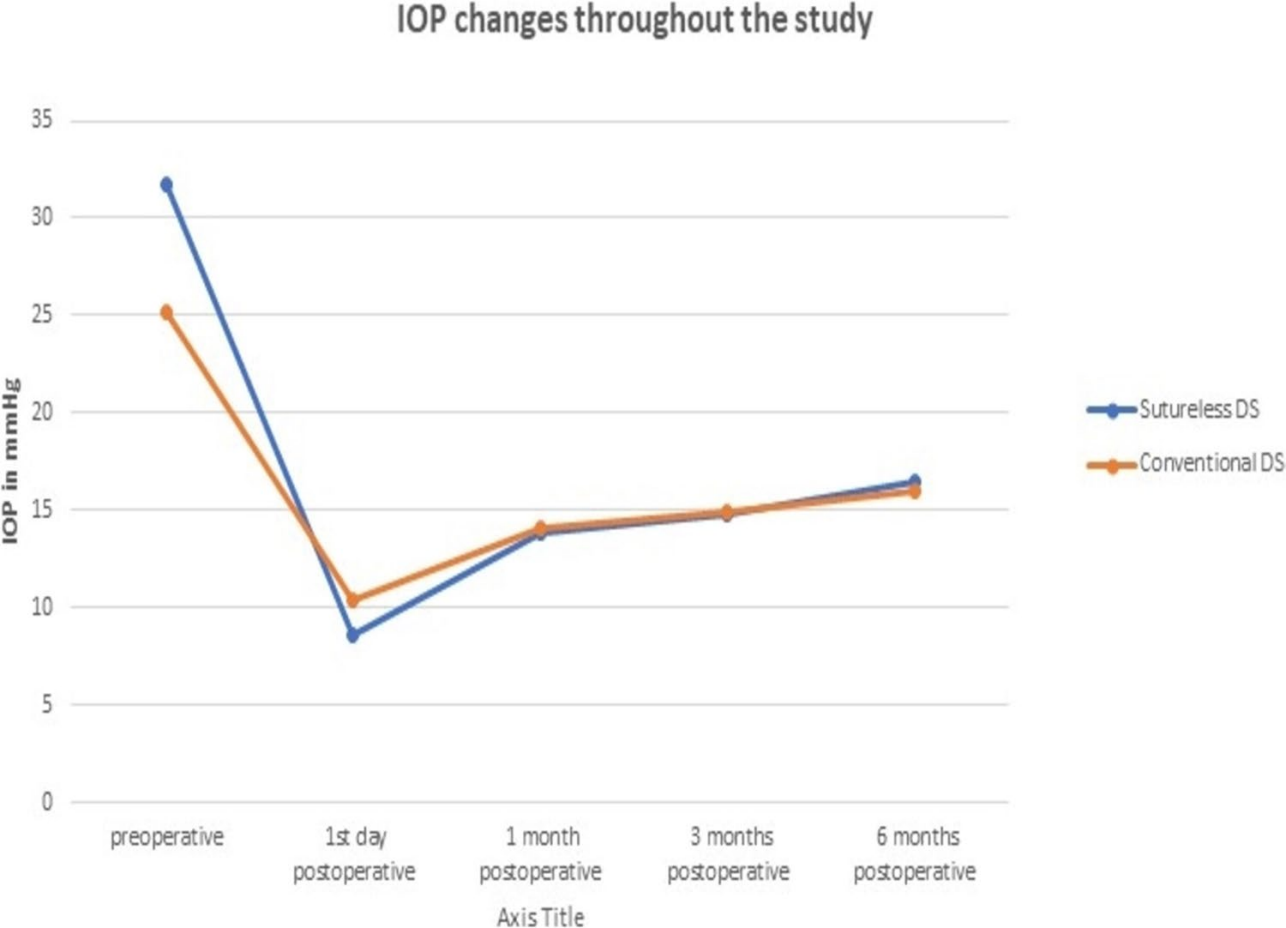
Line chart comparing the changes of IOP between the two groups throughout the study period

Regarding IOP, both surgeries showed significant reduction of IOP all through the study period with no significant difference between both groups. Values of IOP throughout the follow up period are shown in Table 2 and Fig. 2. Also, use of anti-glaucoma medications dropped significantly at 6 months postoperatively in both groups with no significant difference between the 2 groups (Table 2).

**Table 2:** Comparison between the 2 groups regarding changes of IOP and use of anti-glaucoma medications

	Group A				Group B				*P* value comparing the 2 groups
	Mean	SD	Median	*P* value compared to preoperative	Mean	SD	Median	*P* value compared to preoperative	
Preoperative IOP	31.677	8.85	30	< 0.001	25.11	7.35	24.5	< 0.001	0.003
1st day post-op IOP	8.645	3.93	8	< 0.001	10.428	6.5	10	< 0.001	0.082
1-month post-op IOP	13.87	5.95	12	< 0.001	14.107	5.47	13	< 0.001	0.578
3 months post-op IOP	14.83	4.76	14	< 0.001	14.92	4.55	14.5	< 0.001	0.877
6 months post-op IOP	16.45	4.09	17	< 0.001	15.96	4.36	15.5	< 0.001	0.473
Preoperative no. of anti-glaucoma drugs	2.91	1.11	3		2.21	0.42	2		0.001
No. of anti-glaucoma drug at 6 months	0.55	0.83	0	< 0.001	0.54	0.83	0	< 0.001	0.943

Group A: sutureless deep sclerectomy group, Group B: conventional deep sclerectomy group

*IOP* intraocular pressure, *Post-op* postoperatively, *SD* standard deviation

Regarding percentage of reduction of IOP, in group A, mean reduction was 71.37%, 53.35%, 50.30%, and 44.33% at 1st day, 1 month, 3 months, and 6 months, respectively, and in group B, mean reduction was 57.62%, 40.63%, 37.41%, and 31.68% at 1st day, 1 month, 3 months, and 6 months, respectively. Comparison between percentage of reduction in both groups showed no statistically significant difference.

In group A, 20 eyes (64.51%) had IOP ≤ 18 mmHg without use of anti-glaucoma drugs at 6 months postoperatively (complete success), and 8 eyes (25.80%) had IOP ≤ 18 mmHg with the use of anti-glaucoma drugs (qualified success), while this target pressure was not achieved in 3 eyes (9.67%) that were classified as failure. In group B, 23 eyes (79.31%) had IOP ≤ 18 mmHg without use of anti-glaucoma drugs at 6 months postoperatively (complete success), and 4 eyes (13.79%) had IOP ≤ 18 mmHg with the use of anti-glaucoma drugs (qualified success) while this target pressure was not achieved in 2 eyes (6.89%) that were classified as failure.

**Fig. 3:**
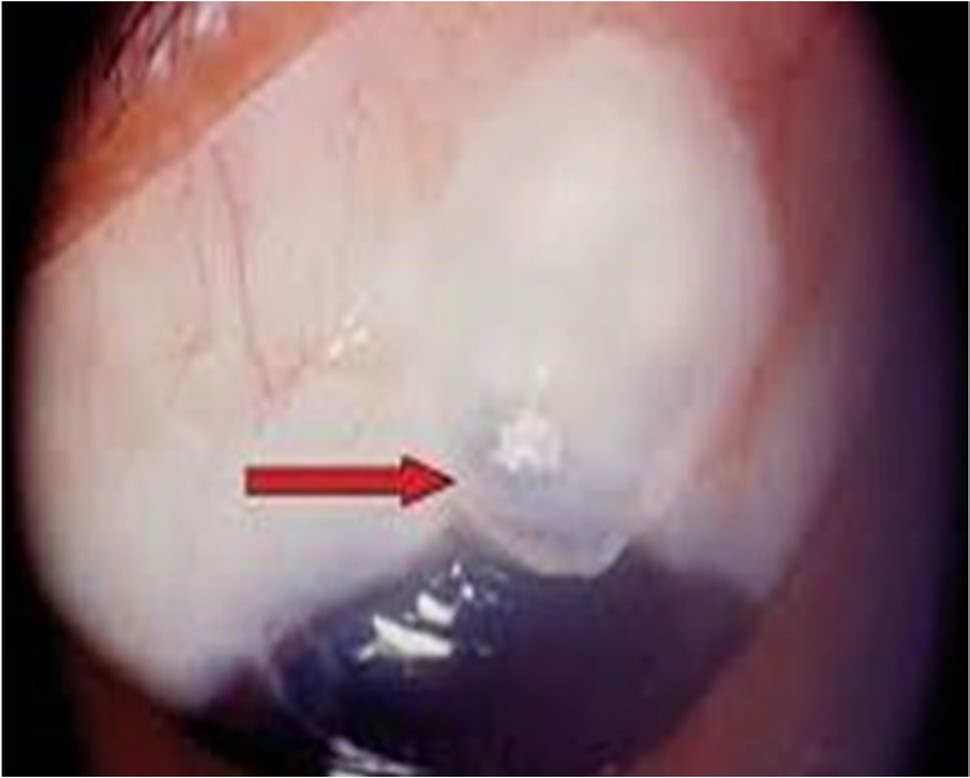
Cystic bleb in one case of group A

Regarding reported complications, in group A, one eye (3.22%) developed cystic bleb (Fig. 3), 2 eyes (6.44%) developed Tenon’s capsule fibrosis, and 1 eye (3.22%) showed bleb failure due to iris adhesions. In group B, one eye (3.44%) developed cystic bleb, one eye (3.44%) developed dellen, and one eye (3.44%) was complicated with conjunctival leak that was managed by conjunctival re-suturing 2 days postoperatively.

**Fig. 4:**
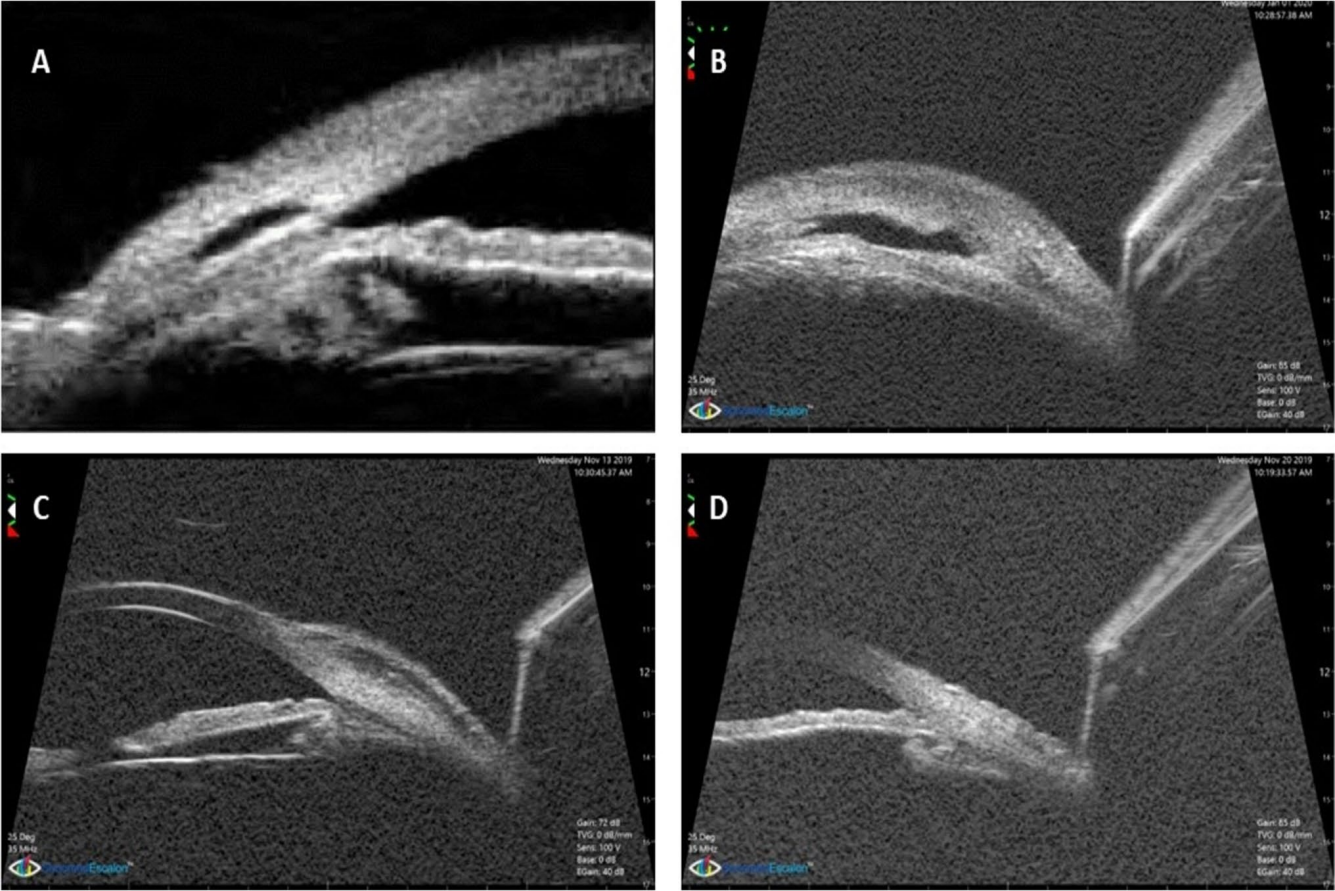
UBM pictures of different cases of the study: **A**, **B** functioning bleb with maintained intrascleral lake in group A and B respectively. **C** Obliterated intrascleral lake with maintained subconjunctival bleb. **D** Non-functioning bleb with obliterated intrascleral and subconjunctival spaces

Analysis of UBM data, 1-month post operatively, 2 blebs were non-functioning with obliterated scleral lake in group A for whom YAG gonio puncture was done. In group B, one patient had an eye that had non-functioning bleb that started using anti-glaucoma drugs. Three months postoperatively, there were 2 additional non-functioning blebs in group A: one of them underwent goniopuncture and the other did needling with mitomycin C. In group B, there was one additional non-functioning bleb, and the patient started medical treatment. At 6 months postoperatively, two additional blebs were non-functioning in group A; one of them showed iris adhesion, and both started medical treatment to control IOP, and one additional non-functioning bleb appeared in group B and the patient started medical treatment to control IOP. Figure 4 shows samples of UBM pictures of functioning and non-functioning blebs.

Collectively, with filtering bleb and preserved scleral lake, mean IOP was found to be around 13 mmHg, and with non-functioning bleb mean IOP found to be around 24 mmHg, suggesting an important role for intrascleral filtration in lowering IOP.

Regarding incidence and timing of interventions, in group A, 5 eyes had YAG goniopuncture done, 2 eyes had goniopuncture and needling, and 5 eyes had needling, and one of them was with mitomycin c. Mean time of interventions was 115 ± 45.80 days postoperatively (range 1.5 to 4 months). In group B, 3 eyes had goniopuncture done, 1 eye had needling, 1 eye had bleb excision, and one had conjunctival re-suturing. Mean time of interventions was 80.33 ± 58.45 days postoperatively (range 1 to 4 months).

## Discussion

In the present prospective randomized interventional study, we compared the short-term IOP-lowering effect of sutureless deep sclerectomy (SDS) to conventional deep sclerectomy. There is one report in literature on short-term results of SDS, so to the best of our knowledge (PubMed search), this is the first study to compare its results to the conventional deep sclerectomy.

Absence of sutures at the scleral edges allows the scleral flap to float freely which permit more aqueous to be drained in the subconjunctival space and help to maintain the presence of intrascleral lake [7].

In SDS group (group A), there was significant IOP reduction with mean preoperative IOP = 31.67 ± 8.85 mmHg, and mean postoperative IOP values were 8.64 ± 3.93, 13.87 ± 5.95, 14.83 ± 4.76, and 16.45 ± 4.09 mmHg at 1st day, 1st month, 3 months, and 6 months postoperatively, respectively (*P* value < 0.001 at all these times). The mean percentage of reduction at 6 months postoperatively was 44.33 ± 20% compared with the preoperative IOP. These results were comparable to Abdelrahman et al.’s results in a series of 24 eyes with follow up for 6 months; the mean preoperative IOP was 31.72 ± 10.71 mmHg, and mean IOP postoperatively was 6.84 ± 1.54, 10.83 ± 2.35, 13.05 ± 4.59, 13.61 ± 3.97, and 15.07 ± 3.22 mmHg at 1st day, 1st month, 3 months, and 6 months, respectively (*P* value < 0.001 at all these times). The mean percentage of reduction at 6 months postoperatively was 47 ± 24.9% compared with the preoperative IOP [7].

In group A, we gained complete success in 20 eyes (64.51%) and qualified success in 8 eyes (25.80%), and there were 3 eyes (9.67%) reported as failure. Abdelrahman et al. reported 71.4% of cases with complete success and 28.6% with qualified success, and no cases were reported as failure [7].

The use of anti-glaucoma drugs decreases significantly after SDS in our study with mean number of medications 2.91 ± 1.11 preoperatively and 0.55 ± 0.83 at 6 months postoperatively (*P* < 0.001). Abdelrahman et al. also reported significant reduction in number of medications with mean number of medications 2.88 ± 1.36 preoperatively and 0.29 ± 0.46 at 6 months postoperatively (*P* < 0.001) [7].

In conventional DS group (group B), there was significant IOP reduction with mean preoperative IOP = 25.11 ± 7.35 mmHg, and mean postoperative IOP values were 10.42 ± 6.50, 14.10 ± 5.47, 14.92 ± 4.55, and 15.96 ± 4.36 mmHg at 1st day, 1st month, 3 months, and 6 months postoperatively respectively (*P* value < 0.001 at all these times). The mean percentage of reduction at 6 months postoperatively was 31.68 ± 25.12% compared with the preoperative IOP. In this group, complete success was achieved in 23 eyes (79.3%), qualified success was achieved in 4 eyes (13.7%), and failure was observed in 2 eyes (6.9%). Our results were comparable to the published results of deep sclerectomy [1, 10–12].

Comparison between results of both groups showed lower IOP values in the 1st postoperative day in group A (mean IOP = 8.64 ± 3.93 mmHg) than group B (mean IOP = 10.42 ± 6.50 mmHg), yet this difference was not statistically significant (*P* value = 0.08). This lower IOP in SDS cases can be attributed to the absence of sutures at the scleral flap edges with less resistance to the flow of aqueous than in conventional DS cases. The values of IOP in both groups came closer to each other with less difference as the time passed postoperatively; this may indicate that the IOP-lowering effect of SDS is better than conventional DS in the early postoperative period.

Having a statistically significant difference between preoperative values in both groups, we better used the percentage of reduction of IOP compared to the preoperative values than depending on the absolute values only. Percentage of reduction of IOP was more in SDS group than in the conventional DS group at all times throughout the follow up period, but the difference was not statistically significant.

The effect of loosening the suture closing at the flap edges in deep sclerectomy on IOP was studied by Rashid et al. after 1-year follow up, and they reported a significant drop of IOP in the loose suture group as compared to the tight suture group. The former group achieved an 18.9 mmHg reduction of IOP as compared to a drop of 12.6 mmHg in the latter group (*P* = 0.045) [13].

We used MMC in its maximum allowable concentration (0.4 mg/ml) for 2 min in both groups to gain the maximum IOP-lowering effect. This was confirmed by Kozobolis et al. who reported better IOP reduction (11.7 mmHg or 42.3%) in DS with MMC compared with 7.1 mmHg or 27.6% in DS without use of MMC (*P* = 0.05) in cases with “POAG after 36 months of follow up [14]. Also, Anand and Atherley reported that the use of MMC decreased the need for YAG goniopuncture (45%) compared to 81% in cases with POAG [15].

Studying the bleb morphology using UBM revealed the absence of intrascleral lake (non-functioning scleral bleb) in 6 eyes in group A and 3 eyes in group B at 6 months postoperatively. Absence of intrascleral lake was accompanied with failure of control of IOP in these cases and the need for use of anti-glaucoma drugs, with mean IOP in non-functioning bleb of 24 mmHg and 13 mmHg in functioning bleb. This was consistent with the results of Elmekawey et al. that found negative correlation between bleb dimensions and postoperative IOP after deep sclerectomy with and without Ologen implant in cases with OAG [2]. On the other hand, Khairy et al. reported that the intrascleral lake was a common finding in their study, but they found no correlation between the bleb dimensions and the outcome of surgery [16].

Regarding postoperative complications, no serious complications were encountered in either group. In the SDS group, 1 eye (3.22%) developed cystic bleb, 2 eyes (6.44%) developed Tenon’s capsule fibrosis, and 1 eye (3.22%) showed bleb failure due to iris adhesions. Abdelrahman et al. reported 1 eye (4.2%) with dellen, 2 eyes (8.3%) with Tenon cysts (8.3%), and 1 eye (4.2%) with conjunctival recession without need for intervention [7]. In group B, 1 eye (3.44%) had cystic bleb, 1 eye (3.44%) had dellen, and 1 eye (3.44%) was complicated with conjunctival leak that needed re-suturing. No serious complications like endophthalmitis or severe hypotony complications were reported in both groups; this was consistent with different DS studies [10–12].

Sutureless deep sclerectomy seems to be a safe and effective modification, with significant IOP reduction in both POAG. It shortens operative time and reduces the cost of sutures without compromising the outcome. The use of MMC enhances its effect, and the non-use of implants helps to reduce the cost. This study is the first to compare this technique to conventional DS; however, it has some limitations like short-term follow up, the relative younger patients’ ages in comparison to the commonly encountered open-angle glaucoma patients and the lack of comparing the surgery duration in minutes, and the exact cost between both groups which can be considered for future study with longer follow up and more homogenous study groups.

## Data Availability

All the data used and/or analyzed during the current study are available and can be presented by the corresponding author upon a reasonable request.
